# Endovascular Intervention in Acute Ischemic Stroke: History and Evolution

**DOI:** 10.3390/biomedicines10020418

**Published:** 2022-02-10

**Authors:** Junaid Ansari, Rachel Triay, Sandeep Kandregula, Nimer Adeeb, Hugo Cuellar, Pankaj Sharma

**Affiliations:** 1 Department of Neurology, Louisiana State University Health Shreveport, Shreveport, LA 71130, USA; Junaid.ansari@lsuhs.edu; 2 School of Medicine, Louisiana State University Health Shreveport, Shreveport, LA 71130, USA; rachel.triay@lsuhs.edu; 3 Department of Neurosurgery, Louisiana State University Health Shreveport, Shreveport, LA 71130, USA; sandeep.kandregula@lsuhs.edu (S.K.); nimer.abushehab@lsuhs.edu (N.A.); Hugo.cuellar@lsuhs.edu (H.C.); 4 Department of Radiology, Louisiana State University Health Shreveport, Shreveport, LA 71130, USA

**Keywords:** mechanical thrombectomy, acute ischemic stroke, modified Rankin scale, stent retriever

## Abstract

Stroke is a leading cause of serious long-term disability in the US. Endovascular therapy (EVT), in the form of mechanical thrombectomy, is now a standard of care for patients with acute ischemic stroke with a large vessel occlusion. This article reviews the evolution of EVT in the management of acute ischemic stroke and how it has led to the concept of tissue window over the widely publicized time window.

## 1. Introduction

Stroke continues to be the second leading cause of death and a leading cause of disability worldwide, despite a substantial decline in age-standardized stroke incidence and mortality in high-income countries compared to low- and middle-income countries [1,2,3]. There is an estimated 2.7% overall stroke prevalence in the United States (US), with projections estimating that by 2030, an additional 3.4 million US adults will have had a stroke, representing 3.9% of the adult population [4]. Intravenous (IV) recombinant human tissue plasminogen activator (r-tPA, alteplase) was approved by US Food and Drug Administration in 1996 [5].

The advent of endovascular thrombectomy (EVT) to manage ischemic strokes has dramatically changed the landscape of stroke management, by increasing functional independence at 90 days and expanding the time window for eligible patients [6]. Furthermore, advanced imaging techniques have introduced the concept of tissue window, which allows for the possibility of selecting the patients for reperfusion therapies based on presence of ischemic penumbra [7]. A series of pivotal clinical trials, published in 2015, demonstrated the benefit of EVT in proximal anterior circulation large vessel occlusion, resulting in a paradigm shift and a new era of acute ischemic stroke therapies [8]. This article will review the evolution and current clinical role of EVT in acute ischemic stroke management.

## 2. Evolution of Endovascular Therapy in Ischemic Stroke 

### 2.1. History

Despite the disproportionately high morbidity and mortality associated with severe ischemic strokes due to large vessel occlusion, there were few therapeutic options available until the advent of intravenous thrombolysis; however, intravenous thrombolysis could be offered to only a few patients, due to the narrow therapeutic window, and strict eligibility criteria and was found to have limited efficacy [5]. These limitations led to an extensive search for alternative and more robust therapies for acute strokes and led to the evolution and development of EVT, for management of acute ischemic stroke. It took almost 75 years for the first clinical trial, MR CLEAN, to demonstrate the safety and efficacy of EVT since the advent of cerebral angiography, in 1927 by Moniz [9].

Among the recent achievements in acute stroke therapies, EVT is one of the most celebrated accomplishments. The first significant attempt to achieve mechanical recanalization was made using pro urokinase. Del Zoppo, Furlan, and others designed the Prolyse in Acute Cerebral Thromboembolism (PROACT,1 and 2) trials to demonstrate the safety, recanalization efficacy, and clinical benefit of IA r-pro-UK in patients with middle cerebral artery (MCA) occlusion, treated within 6 h of stroke onset [10,11].

### 2.2. Early Clinical Trials

The initial clinical trial PROACT-I (Prolyse in Acute Cerebral Thromboembolism), published in 1998, demonstrated that intraarterial administration of 6 mg of pro-urokinase in patients with middle cerebral artery (MCA) M1 and M2 occlusion resulted in higher recanalization rates [11,12]. This was then followed by PROACT-II, where the trial was focused on MCA occlusion and stratified patients based on baseline stroke severity. Eligible patients were randomized to receive 9 mg of IA r-pro-UK over 2 h, plus intravenous (IV) heparin or IV heparin alone. Out of 180 patients randomized, 121 received r-pro-UK, and 59 patients received IV heparin. This study demonstrated a recanalization rate of 66% in the treatment group vs. 18% in the control group. (*p* < 0.001). 40% of r-pro-UK patients and 25% of control patients achieved modified Rankin scale of less than 2. Intracranial hemorrhage within 24 h occurred in 35% of the r-pro-UK group and 13% of the control group. The rate of procedural complication was 1% [13].

Though PROACT-II demonstrated procedural safety, along with better clinical outcome and recanalization rate, there was an increased risk of symptomatic intracranial hemorrhage. However, PROACT-II created a lot of interest amongst stroke neurologists, neurointerventionalists, and device companies, to further look into the role of EVT in acute ischemic stroke due to large vessel occlusion, which led to the development of first-generation stent retrievers. 

Dr. Gobin and others developed the Mechanical Embolus Removal in Cerebral Ischemia (MERCI) retriever at UCLA, and Dr. Duckwiler performed the first case at UCLA in 2001. This case was a success, and they were able to achieve complete recanalization. The MERCI Retriever (Concentric Medical, CA, USA) was the first device approved by FDA for thrombectomy in acute ischemic stroke [14].

MERCI trial reported a recanalization rate of 48% of intracranial blood vessels, which was a significant improvement over the spontaneous recanalization rate of 18% reported in PROACT-II trial. Procedural complications occurred in 13% of patients, and symptomatic ICH occurred in 7.1% of patients. However, this was less than the reported rate of symptomatic hemorrhage in the PROACT trial. Multivariate analysis from MERCI trial showed that recanalization was associated with improved outcome and functional independence at 90 days. Therefore, MERCI was the first significant step towards mechanical recanalization. Although the MERCI trial showed improved recanalization rate, mortality and complication rates were high, leading to the search for safer and efficacious alternatives. 

MERCI retrieval was followed by penumbra revascularization system; in this technique, the penumbra reperfusion catheter was advanced to the proximal edge of the thrombus, the aspiration catheter was then connected to the aspiration pump. This pump generated pressure of −20 inches Hg. Thereafter, a continuous aspiration and debulking process ensued using the Penumbra Separator. Subsequently, extraction was then performed using the thrombus extracting ring, which was deployed under proximal flow arrest [15]. The Penumbra Pivotal Stroke trial reported a recanalization rate of 81%, the procedural event rate was 12.8%, out of which 2.4% were serious events. The symptomatic ICH rate was 11%. Patient neurological recovery and the functional outcome showed improvement; 25% of patients were reported to have National Institutes of Health Stroke Scale (NIHSS) scores of 0 to 1 or improvement of more than 10 points on the NIHSS.

The reported 90-day modified Rankin Scale (mRS) score was 27%, which was comparable to the MERCI 2 trial; however, considering the stunning recanalization rate, this was lower than expected. Therefore, this trial highlighted that patient selection is as crucial as reperfusion.

Thereafter, in 2013 SYNTHESIS, MR RESCUE, and Interventional Management of Stroke-III (IMS-III) were published.

The Mechanical Retrieval and Recanalization of Stroke Clots Using Embolectomy (MR RESCUE) trial was a randomized, controlled, multicenter trial in which patients with acute stroke presenting with NIHSS of 6–29 who had a large vessel occlusion were recruited within 8 h of onset. All patients underwent pretreatment multimodal CT or MRI to detect the presence of penumbra [16]. 

The neural tissue which is ischemic but has not yet sustained irreversible damage is the target for all acute therapies aimed at revascularization. One of the well-developed techniques for imaging ischemic penumbra in acute ischemic stroke patients is combined diffusion and perfusion imaging. Penumbra was defined as neural tissue which demonstrated decreased perfusion but in which the cytotoxic abnormalities and diffusion restriction have not yet developed. 

MR RESCUE investigators used MERCI retriever for embolectomy. There was no difference in the mean mRS between embolectomy and standard care (3.9 vs. 3.4; *p* = 0.23). Similarly, in patients with no penumbra, embolectomy was not superior to standard care (mean score, 4 vs. 4.4; *p* = 0.32). The rate of symptomatic intracranial hemorrhage was 4%.

MR RESCUE was a disappointment for the stroke community; however, there were some encouraging findings. Firstly, proper selection of patients based on imaging led to decreased rate of symptomatic hemorrhage, and more importantly, the mean mRS was better in embolectomy group than the standard care group despite the low rate of recanalization in the embolectomy group, which was probably due to use of first-generation embolectomy devices. Subgroup analysis did show that mean mRS at 90 days was 3.5 in patients who underwent partial or complete recanalization, whereas it was 4.4 in patients who did not undergo revascularization [16]. 

### 2.3. Second Generation Thrombectomy Devices

**Figure 1:**
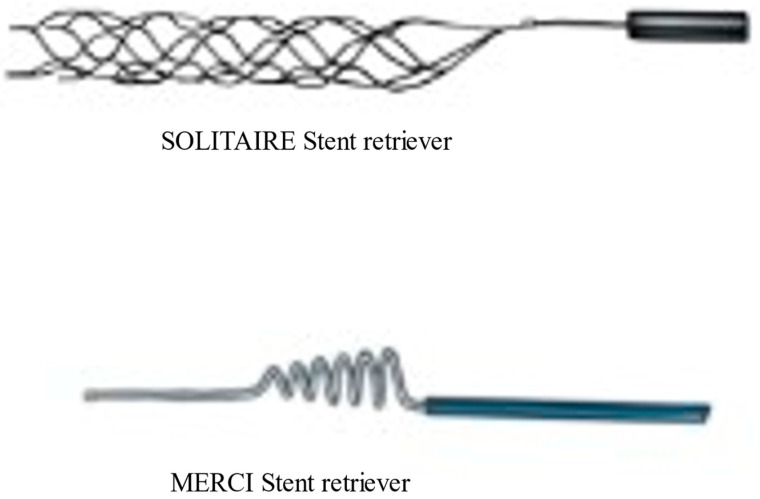
Newer generation solitaire stent retriever and First generation MERCI stent retriever.

The evolution of more sophisticated and advanced thrombectomy devices has continued, alongside thrombectomy techniques and imaging technology. The first retrievable stent introduced was Solitaire (Medtronic, Irvine, CA, USA), (Figure 1); this was a fully retrievable and self-expanding intracranial stent [17]. The design of this stent was first described in 2003 [18]. The first case report of successful thrombectomy using Solitaire stent retriever was published in 2009 [16]. With Solitaire, the successful recanalization rate as determined by the core laboratory in the SWIFT trial was 69%, compared to 30% for the MERCI retriever. However, this recanalization rate was lower than the recanalization rates of 88–92% reported in open series [17]. Good neurological outcomes at 90 days were seen more often in the Solitaire group than in the MERCI group. Successful recanalization without symptomatic intracranial hemorrhage was 61% in Solitaire group as compared to 24% in the MERCI group. Overall, the newer generation, Solitaire stent retriever was safer and was associated with improved neurological outcomes [17].

SYNTHESIS and IMS-III were two major clinical trials that were published in 2013, but to the despair of the stroke community, both demonstrated that endovascular treatment was not superior to the standard care at that time [18,19]. Patients between 18 to 80 years presenting with ischemic stroke within 4.5 h, in whom intracranial hemorrhage had been ruled out, were included in SYNTHESIS trial. Both intraarterial pharmacologic thrombolysis and mechanical thrombolysis were permitted. Pre-procedure vascular imaging and perfusion imaging were not required for enrollment in these trials [18,19]. In the SYNTHESIS and IMS-III trial proportion of patients who had symptomatic intracranial hemorrhage was 6%. This was almost equivocal to the intravenous thrombolysis group. This trial, along with IMS-III, was one of the first to demonstrate the safety of EVT. This trial’s major limitations were limited use of advanced stent retriever, lack of vascular and perfusion imaging for patient selection. 

IMS-III was also limited by the lack of vascular and perfusion imaging for patient selection. In the latter half of the trial, the protocol was amended to allow use of CT angiography for patient selection. Subgroup analysis of IMS-III did report marginal benefit with IA therapy in CTA-positive patients. Also, newer generation stent retrievers were used in a small percentage of patients recruited in IMS-III trial. The time to EVT in IMS-III was 32 min longer than IMS-I trial, which may be one of the major reasons for lack of benefit as 30 min delay in EVT is associated with 10% decrease in the probability of functional independence [20].

Despite these setbacks, several noteworthy points emerged out of IMS-III trial. Firstly, endovascular therapy was associated with high rate of recanalization, the rate of partial or complete recanalization at 24 h was 81% for an occlusion in the internal carotid artery, 86% for an MCA M1 occlusion. The rates in the intravenous t-PA group were 35% for an occlusion in the internal carotid artery and 68% for an MCA M1 occlusion. In addition, the proportion of patients with mRS of less than 2 at 90 days was 71% in patients who achieved complete reperfusion (TICI 3), whereas only 12.7% of patients in whom no reperfusion was achieved (TICI 0) had mRS of less than 2 [19].

### 2.4. Modern Clinical Trials and Paradigm Shift

**Figure 2:**
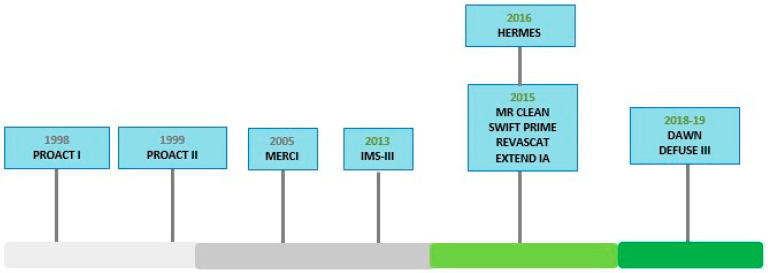
Clinical Timeline for endovascular thrombectomy trials.

Important lessons learned from these trials were utilized to design subsequent trials and in 2015 the outlook was completely changed with publication of 5 different clinical trials with better research design including MR CLEAN [21], ESCAPE [22], SWIFT PRIME [23], REVASCAT [24], and EXTEND IA [25] (Figure 2) (Table 1).

MR CLEAN included patients with ischemic strokes within 6 h of onset who had occlusion of intracranial internal carotid artery (ICA), proximal MCA or proximal anterior cerebral artery. The patients were randomized to standard care and endovascular therapy. Retrievable stents were used in 85% of patients. The absolute difference between the proportion of patients who were functionally independent (mRS of less than 2) was 13.5% in favor of endovascular group. MR CLEAN reported reperfusion rate of 58%. Symptomatic ICH rate was 7.7% [21]. This was the first breakthrough that demonstrated the benefit of EVT for anterior circulation ischemic stroke. 

Following the publication of MR CLEAN early interim analysis was conducted for all five other major clinical trials. To their credit, the interim analysis was positive for all these major trials. 

**Figure 3:**
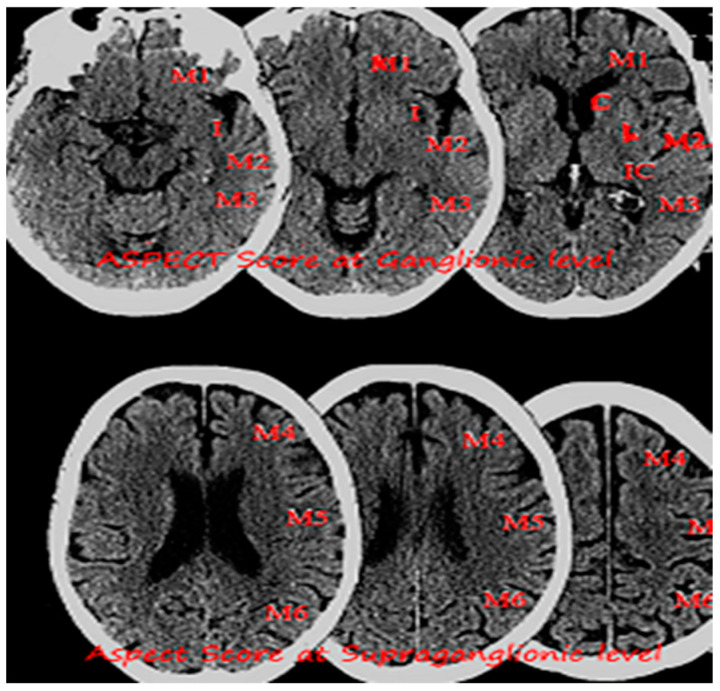
Image showing non-enhanced CT head with an Alberta Stroke Program Early CT Score (ASPECTS) at Ganglionic and Supra ganglionic level.

The ESCAPE trial included patients with NIHSS of more than 6 and utilized advanced imaging based on The Alberta Stroke Program Early CT Score ASPECTS [26] and collateral circulation assessment. ASPECTS is a 10-point scoring system to quantify early ischemic changes in the middle-cerebral-artery territory, with a score of 10 indicating normal and 1 point subtracted for each abnormal region (Figure 3). Revascularization rate was 72.4%. Interim analysis reported 23.7% absolute difference between proportion of patients who were functionally independent with odds ratio of 2.6 in favor of endovascular therapy. Notably, the rate of symptomatic ICH was 3.6% in the intervention group. ESCAPE data suggested that better patient selection based on ASPECTS and collateral circulation not only leads to better outcomes but also improved safety profile. 

SWIFT PRIME included patients within 6 h of onset, perfusion imaging and ASPECTS were used to identify patients who had small core. This trial had homogenous design and newer generation stent retrievers were invariably used for thrombectomy of proximal segment of middle cerebral and intracranial segment of ICA. SWIFT PRIME reported impressive recanalization rate of 88.0% [23]. Because 60.2% patients in SWIFT PRIME achieved functional independence, which was significantly higher than 33% reported in MR CLEAN and comparable to the 71% reported in EXTEND IA [25]. Interestingly both SWIFT PRIME and EXTEND IA reported symptomatic ICH rate of 0% further reinforcing the fact that advanced imaging was helpful in improving the safety profile of revascularization procedure.

All these trials underwent a rigorous meta-analysis in the HERMES collaboration revealing EVT to be beneficial to most patients with large vessel proximal anterior circulation occlusion. In the HERMES trial prespecified primary outcome was the degree of disability on the mRS at 90 days. HERMES pooled 1287 participants (634 EVT, interventional population and 653 standard medical treatment). It showed that mRS score 0–2 at 90 days was higher in the intervention arm compared to standard medical therapy. There was no significant difference in mortality and risk of intraparenchymal hematoma type 2 and symptomatic intracranial hemorrhage between intervention and control populations [8]. NIHSS was higher in control population (mean 14.2) compared to intervention population (mean 10.4) at 24 h post ictus. Similar trends were noted in the NIHSS change from baseline to 24 h (mean −6.4 interventional vs. mean −2.6 control). HERMES confirmed the benefit of EVT in a wide range of subgroups, including the elderly population, and patients not receiving tPA. Number needed to treat in the HERMES collaboration study was 2.6.

**Figure 4:**
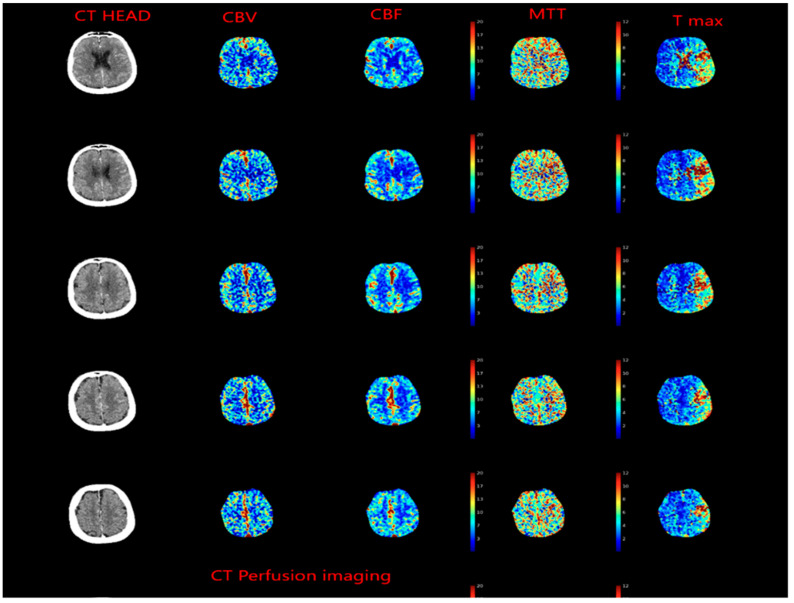
CT Perfusion imaging in patient presenting with left middle cerebral artery occlusion, cerebral blood volume (CBV) is preserved, whereas cerebral blood flow (CBF) is decreased, and mean transit time (MTT) is increased.

Meanwhile, evolving imaging techniques were helpful in evaluating penumbra, which was defined as tissue which is ischemic but is still viable (Figure 4). This gave raise to concept of “tissue window” which was later utilized in DAWN [27] and DIFFUSE [28] trials. 

### 2.5. Evolution of the Concept of the Tissue Window and Late Window Trials

Dawn recruited patients within 6 to 24 h and divided them into three groups based on age and core volume. In addition, clinical and core mismatch were assessed on perfusion or diffusion-weighted imaging (DWI). Patients were then randomized to standard care vs. EVT; the rate of functional independence was 49% in the EVT group and 13% in the control group, and utility-weighted modified Rankin scale at 90 days was 5.5 in the thrombectomy group compared to 3.4 in the control group [27]. Interestingly, the benefit of EVT was consistent in older patients and in the cohort with large core. Another notable feature was 13% functional independence reported in control group which was worse than 26% reported in pooled analysis. 

The DEFUSE 3 [28] trial included patients with ischemic core volume of less than 70 mL and the salvageable brain (penumbra) volume more than 15 mL, a ratio of volume of ischemic to infarct volume of 1.8 or more. The trial reported favorable distribution of disability based mRS in the EVT group. In addition 45% had functional independence in the EVT group compared to 17% in the medical therapy group. Therefore, both trials demonstrated remarkable benefit of reperfusion in patients presenting late with salvageable brain tissue.

The rate of symptomatic intracranial hemorrhages did not differ significantly between the thrombectomy and medical therapy in both trials. The rate of symptomatic ICH was higher in the large core infarct volume, although not statistically significant (9% vs. 5%, *p* = 0.391). The death rate was also similar (18% vs. 20%, *p* = 0.799). 

## 3. Conclusions

The American Heart Association and the European Stroke Organization recommended EVT, in addition to the best medical management, in adults with an anterior circulation stroke, presenting within 6 h of symptom onset and meeting the following criteria: pre-stroke mRS of less than 2, causative occlusion of internal carotid artery or middle cerebral artery, age > 18 years, ASPECTS and NIHSS of more than 6 [29,30]. There is still a lot of debate whether EVT should be considered in patients who present with NIHSS of less than 6. In patients who presented after 6 h of onset and met the criteria of the DAWN and DIFFUSE 3 trials, EVT is recommended. EVT is currently not recommended for patients with large core and low ASPECTS; however, a multicenter randomized clinical trial, TESLA, is enrolling patients with low ASPECT scores.

**Figure 5:**
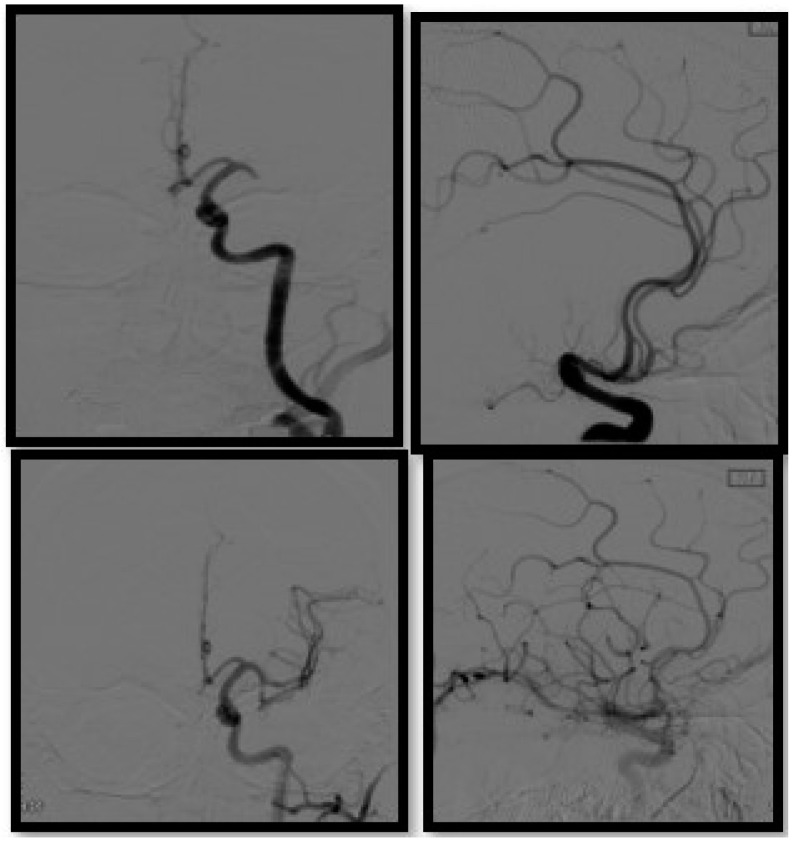
Diagnostic cerebral angiography in a patient presenting with left MCA occlusion, AP and lateral view pre (**upper** row) and post thrombectomy (**bottom** row).

**Figure 6:**
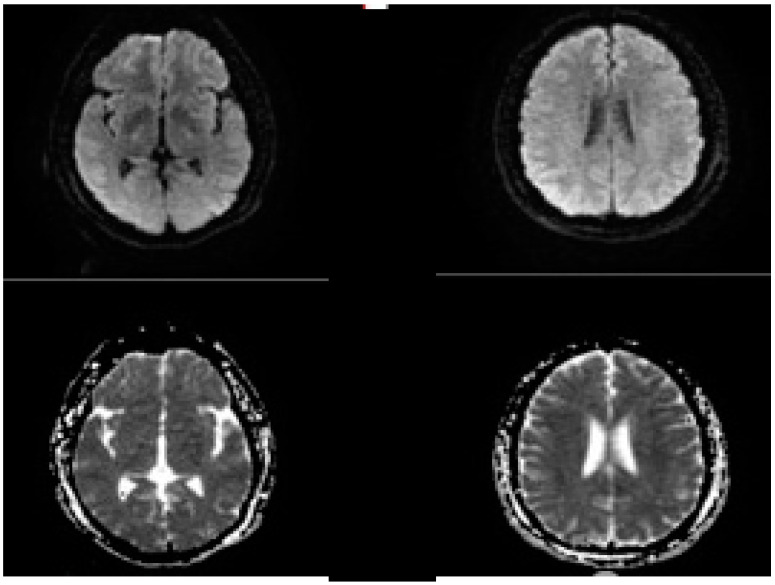
Post procedure of MRI brain in the same patient presenting with left MCA occlusion, no DWI lesions were seen.

We recommend that patient selection is key to good clinical outcome. Vascular imaging of the head and neck is needed to look for large vessel occlusion, status of leptomeningeal collaterals and circle of Willis; perfusion imaging is important for patient selection (Figure 5 and Figure 6). Perfusion imaging can further provide useful information about penumbra and is particularly useful for patients presenting late.

DAWN and DIFFUSE 3 demonstrated the strong benefit of thrombectomy in appropriately selected patients, presenting after six hours. Although the notion “time is brain” was critical in establishing stroke workflow and increasing awareness for strokes, recently we have seen the emergence of the novel concept, “tissue is brain”, which is equally important in appropriate patient selection. 

## 4. Future Directions

The role of EVT for posterior circulation stroke should be evaluated further, with carefully and well-designed clinical trials. The recently published BASICS trial showed that among patients with stroke from basilar artery occlusion, endovascular therapy and medical therapy did not differ significantly with respect to a favorable functional outcome; however, as reflected by the wide confidence interval for the primary outcome, the results of this trial may not exclude the potentially substantial benefit of endovascular therapy [31].

At our center, we do recommend EVT to patients presenting with occlusion of the basilar artery and a proximal segment of the posterior cerebral artery. Several other studies are evaluating the role of neuroprotective agents for preserving ischemic neural function.

## Figures and Tables

**Table 1:** Summary of major clinical trials.

TRIAL	Inclusion Criteria	Number of Patients	Mean/Median Age	Median NIHSS	IV tPA	Re Perfusion	sICH	mRS Less than 2 at 90 Days	limitation	Mortality 90 Days
MR CLEAN	NIHSS > 2, occlusion of vessel imaging	500 I: 233 C:267	I:65.8 C65.7	I: 17 C: 18	I: 87.1 C:90.6	58.7	I 7.7 C6.4	I 32.6 C 19.1	No perfusion imaging. Low reperfusion rate	I:18.9 C:18.4 At 30 days
ESCAPE	NIHSS > 5, ASPECT > 6, collateral assessment, stent retriever in 84%	315 I 165 C150	I 71 C 70	I 16 C 17	I 72.7 C 78.7	72.4%	I 3.6 C2.7	I 53 C29.3	No perfusion imaging	I: 10.4 at 90 days C 19%
EXTEND IA	Age > 18 y Collateral assesment, perfusion imaging	70 I 35 C 35	I 78.6 C 70.2	I 17 C 13	I 100 C 100	86%	I 0 C 6	I 71 C 40	Small sample size	I 9% C 20%
SWIFT PRIME	NIHSS 8–29 ASPECT > 6 Perfusion imaging	196 I 98 C 98	I 65 C 66.3	I 17 C 17	I 100 C 100	83%	I 0 C 3	I 16 C 35	All patients received IVT	I 9 C12
REVASCAT	NIHSS > 6 ASPECT > 6 AGE 18–85 years	206 I 103 C 103	I 65.7 C 67.2	I 17 C 17	I 68 C 77	65.6%	I 4.9 C1.9	I 43.7 C28.2	Low reperfusion rate	I: 18.4% C:15.5%
DAWN	NIHSS > 10 Core and clinical mismatch Up to 24 h	206 I 107 C 99	I 69.4 C 70.7	I 17 C 17	I 5 C 13	84%	I 6 C 3	I 49 C 13	Severe stroke, Difference in baseline variables	I 19 C 18
DIFFUSE 3	Perfusion with mismatch 6–16	182 I 92 C 90	I 70 C 71	I 16 C 16	I 11 C 9	76%	I 7% C 4%	I 45% C 17%	Patient papulation is older.	I 14% C 26%

## Data Availability

Not applicable.

## Abbreviations

1	PROACT: Prolyse in Acute Cerebral Thromboembolism
2	MERCI: Mechanical Embolus Removal in Cerebral Ischemia
3	MR RESCUE: Mechanical Retrieval and Recanalization of Stroke Clots Using Embolectomy
4	IMS-III: Interventional Management of Stroke-III
5	mRS: Modified Rankin Scale
6	SYNTHESIS: Systemic intravenous (IV) thrombolysis with Alteplase in acute ischemic stroke
7	MR CLEAN: Multicenter Randomized Clinical trial of Endovascular treatment for Acute ischemic stroke in the Netherlands
8	ESCAPE: Endovascular Treatment for Small Core and Anterior Circulation Proximal Occlusion with Emphasis on Minimizing CT to Recanalization Times
9	SWIFT PRIME: Solitaire with the Intention for Thrombectomy as Primary Endovascular Treatment
10	REVASCAT: Randomized Trial of Revascularization with Solitaire FR Device versus Best Medical Therapy in the Treatment of Acute Stroke Due to Anterior Circulation Large Vessel Occlusion Presenting within Eight Hours of Symptom Onset
11	EXTEND IA: Extending the Time for Thrombolysis in Emergency Neurological Deficits—Intra-Arterial
12	HERMES: Highly Effective Reperfusion evaluated in Multiple Endovascular Stroke Trials
13	DAWN: DWI or CTP Assessment with Clinical Mismatch in the Triage of Wake-Up and Late Presenting Strokes Undergoing Neurointervention with Trevo
14	DEFUSE: Endovascular Therapy Following Imaging Evaluation for Ischemic Stroke
